# Supplementary material for: Balancing selection and the functional effects of shared polymorphism in cryptic Daphnia species
Source: bioRxiv. 2024 Apr 20:2024.04.16.589693. Preprint. [Version 1] doi: 10.1101/2024.04.16.589693 (PMC11042267; doi:10.1101/2024.04.16.589693)
Supplement: Supplement 1 [file NIHPP2024.04.16.589693v1-supplement-1.pdf]

bioRxiv preprint doi: https://doi.org/10.1101/2024.04.16.589693; this version posted April 20, 2024. The copyright holder for this preprint (which was not certified by peer review) is the author/funder, who has granted bioRxiv a license to display the preprint in perpetuity. It is made available under aCC-BY 4.0 International license.

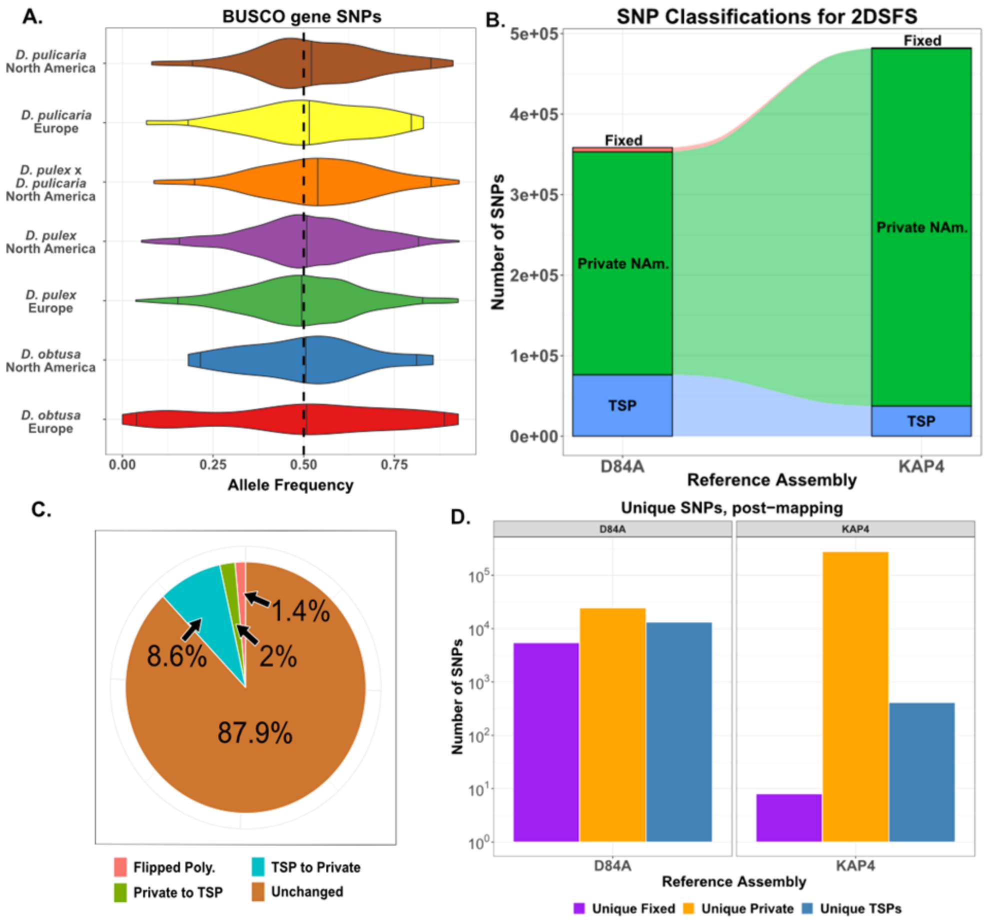

<u>Supplemental Figure 1.</u> No evidence of reference allele bias across the *Daphnia pulex* species complex. A) For each species, we extracted representative individuals (ranging from n=5-100 depending on the number of samples per species) and 1,000 biallelic heterozygous BUSCO gene SNPs (100 bootstraps) to gauge the severity of reference allele bias across the genome. We calculated the proportion of the alternative and reference dosage within a given individual for each site. The x-axis measures the proportion of alternative to reference dosage for each SNP and we show the 95% quantiles and median. **B)** Alluvial plot of the SNP classifications between assemblies of the European *D. pulex* (D84A) and the North American *D. pulex* (KAP4). **C)** Proportion of SNP classification changes when mapping to KAP4 exclusively. **D)** The number of classified SNPs that are exclusive to each assembly.

bioRxiv preprint doi: https://doi.org/10.1101/2024.04.16.589693; this version posted April 20, 2024. The copyright holder for this preprint (which was not certified by peer review) is the author/funder, who has granted bioRxiv a license to display the preprint in perpetuity. It is made available under aCC-BY 4.0 International license.

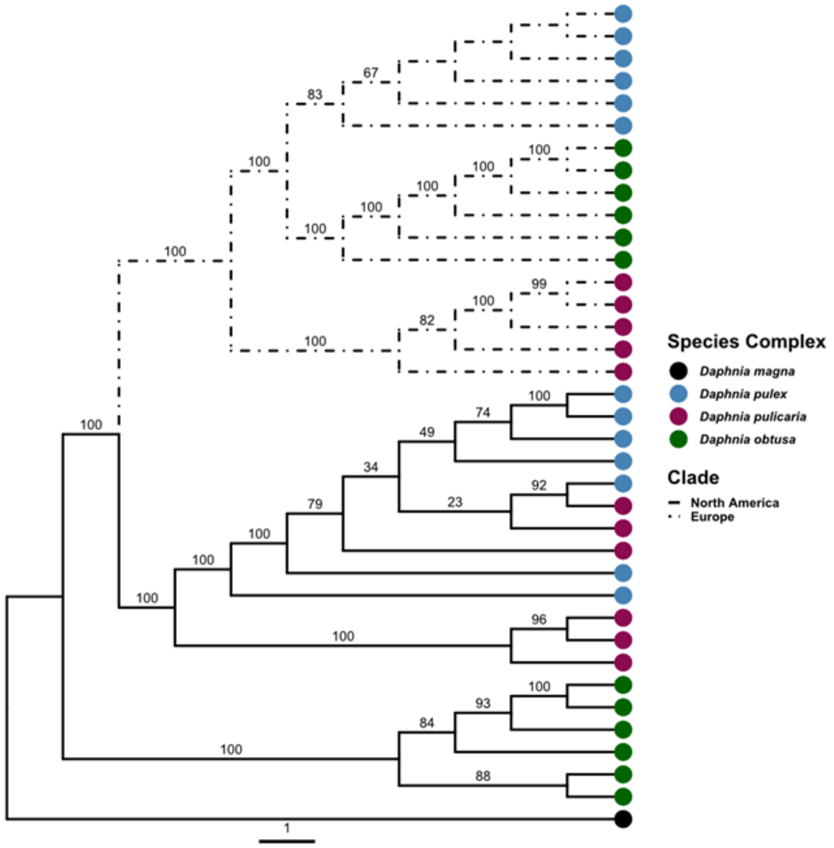

<u>Supplemental Figure 2.</u> Mitochondrial protein-coding tree for the *D. pulex* species complex. A maximum-likelihood tree with the "TN+F+I+G4" model output from IQTree2. The tree is rooted with *D. magna* as an outgroup. Bootstrap supports are listed as node labels.

bioRxiv preprint doi: https://doi.org/10.1101/2024.04.16.589693; this version posted April 20, 2024. The copyright holder for this preprint (which was not certified by peer review) is the author/funder, who has granted bioRxiv a license to display the preprint in perpetuity. It is made available under aCC-BY 4.0 International license.

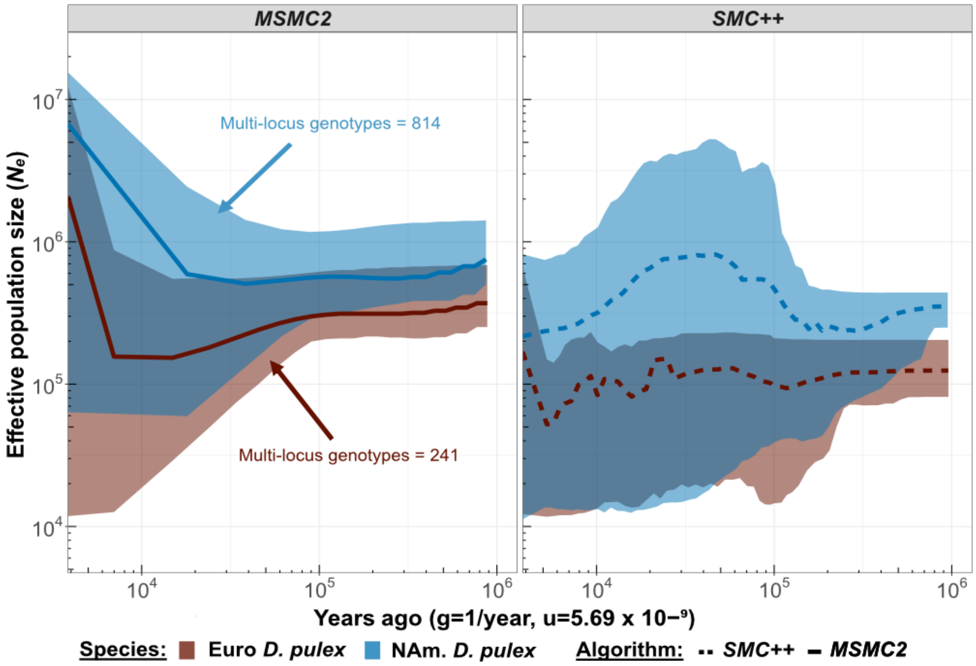

<u>Supplemental Figure 3.</u> Demographic reconstruction of North American and European *D. pulex* species. MSMC2 and SMC++ output for each multi-locus genotype sample. Each multi-locus genotype sample was run independently. The shaded ribbon shows the upper 95% quantiles and lower 5% quantiles from the run estimates.

bioRxiv preprint doi: https://doi.org/10.1101/2024.04.16.589693; this version posted April 20, 2024. The copyright holder for this preprint (which was not certified by peer review) is the author/funder, who has granted bioRxiv a license to display the preprint in perpetuity. It is made available under aCC-BY 4.0 International license.

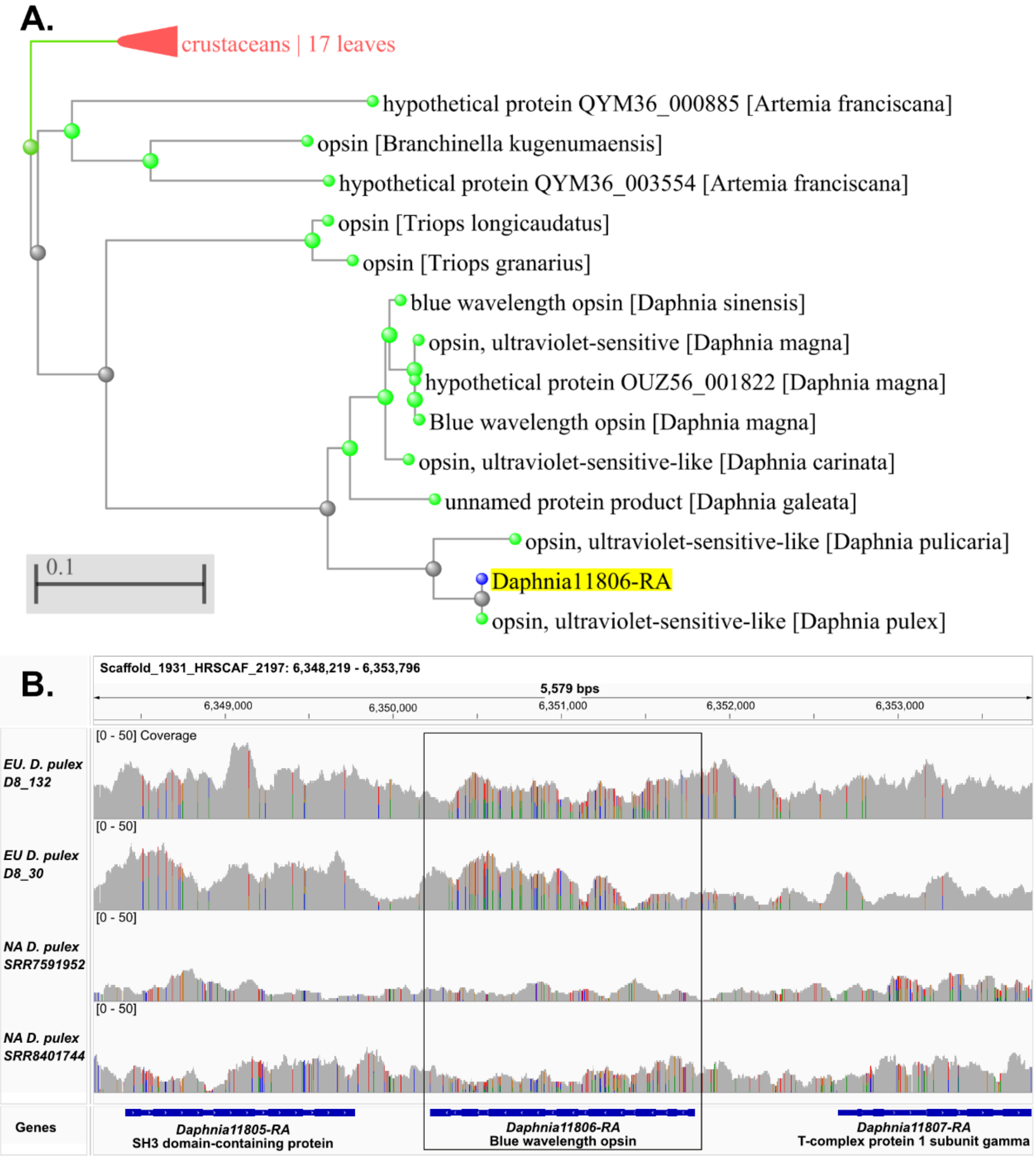

<u>Supplemental Figure 4.</u> Blue wavelength opsin gene and orthologous proteins in Crustacea and within-species heterozygosity. A) This neighbor-joining protein tree was generated using *Blast's* tree widget. The query sequence is highlighted in yellow and has a blue tip symbol. The green tip symbols are related Crustacean protein sequences with the species name in brackets. **B)** We subsampled two representative individuals within the European (EU) and North American (NA) *D. pulex* species and are showing the coverage for each individual set to [0-50]. The vertical-colored bars are heterozygous regions (i.e., split-colored bars) and homozygous alternative alleles (i.e., whole-colored bars), gray base pairs are the reference allele.

bioRxiv preprint doi: https://doi.org/10.1101/2024.04.16.589693; this version posted April 20, 2024. The copyright holder for this preprint (which was not certified by peer review) is the author/funder, who has granted bioRxiv a license to display the preprint in perpetuity. It is made available under aCC-BY 4.0 International license.

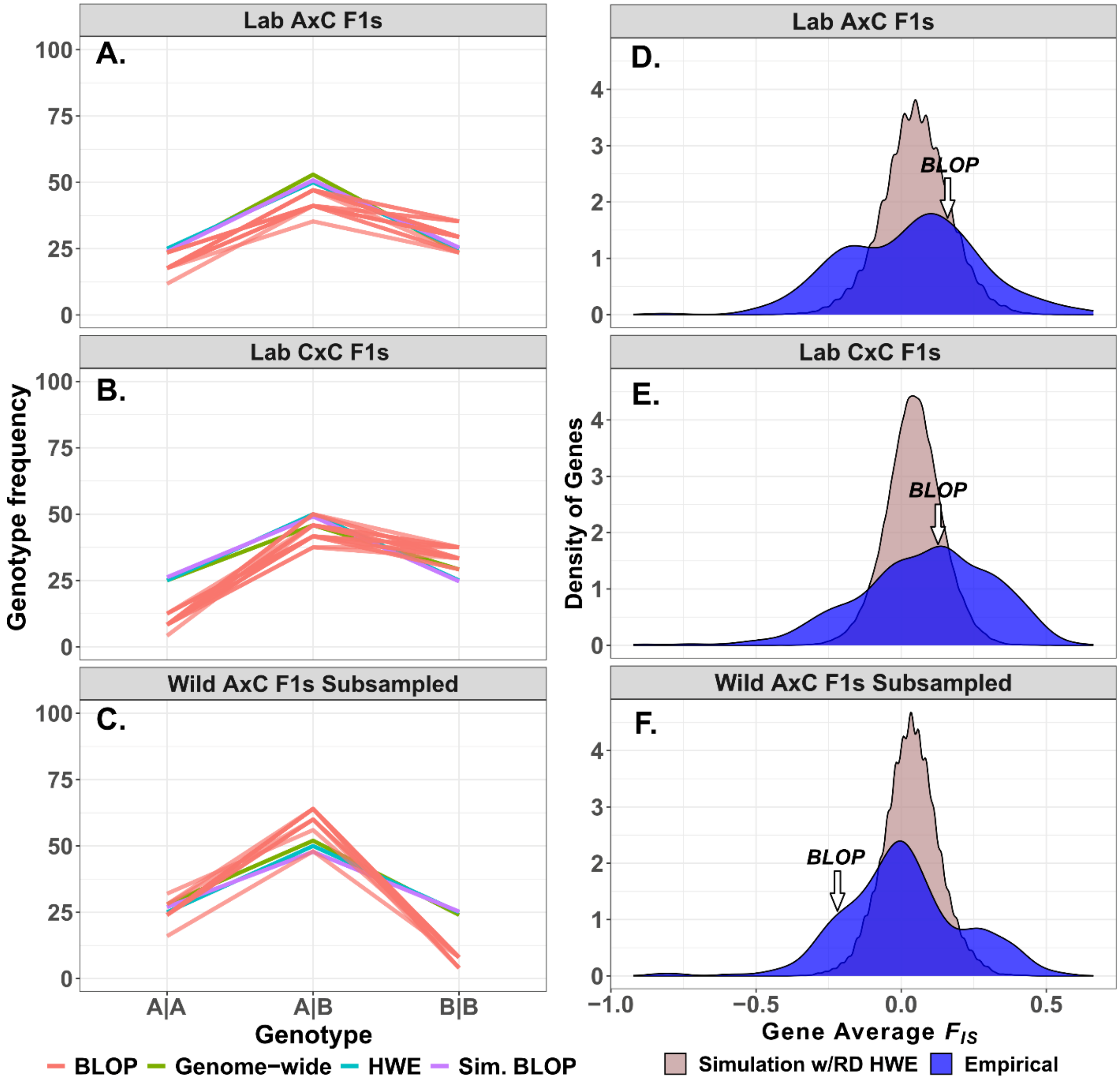

<u>Supplemental Figure 5.</u> Segregation patterns and F_{IS} of polymorphisms across lab and wild crossed *Daphnia* clones. A-C) Average segregation frequency of F1 genotypes expected based on a double heterozygous cross (i.e., AB x AB) using empirical read depth at each SNP. We produced crosses of AxC in the lab shown in panel A and CxC shown in panel B. Panel C shows the F1 genotypes subsampled based on their status belonging to superclones identified in Barnard-Kubow et al. 2022, reflecting a conservative sampling approach. “Genome-wide” is the segregation for SNPs based on the read depth. “HWE” is the segregation pattern expected for Hardy Weinberg equilibrium. “Sim. BLOP” is the segregation pattern expected for the SNPs within the blue opsin gene based on empirical read depth. “BLOP” is the empirical segregation of trans-specific polymorphisms within the blue wavelength opsin gene among F1 genotypes. **D-F)** Distribution of average gene F_{IS}. “HWE Simulation w/RD” is the expected F_{IS} for each gene based on the empirical read depth for each SNP within every gene and “Empirical” is the average is the F_{IS} across genes. The small arrow denotes where the gene average for the blue wavelength opsin falls along the empirical distribution.
